# Blue light stimulation of the blind spot in human: from melanopsin to clinically relevant biomarkers of myopia

**DOI:** 10.1186/s42234-024-00159-0

**Published:** 2024-11-04

**Authors:** Ana Amorim-de-Sousa, Ranjay Chakraborty, Michael J. Collins, Paulo Fernandes, José González‑Méijome, Jens Hannibal, Hosein Hoseini-Yazdi, Scott A. Read, Jens Ellrich, Tim Schilling

**Affiliations:** 1 Clinical & Experimental Optometry Research Lab, Physics Center of Minho and Porto Universities, University of Minho, Braga, Portugal; 2 Myopia and Visual Development Lab, Flinders University College of Nursing and Health Sciences, Bedford Park, South Australia Australia; 3 Contact Lens and Visual Optics Laboratory, Centre for Vision and Eye Research, Optometry and Vision Science, Queensland University of Technology, Brisbane, Australia; 4 Department of Clinical Biochemistry, Bispebjerg and Frederiksberg Hospital, Institute of Clinical Medicine, Faculty of Health Sciences, University of Copenhagen, Copenhagen, Denmark; 5 Dopavision GmbH, Pfuelstrasse 5, 10997 Berlin, Germany; 6 Medical Faculty, Friedrich-Alexander-University Erlangen-Nuremberg, Erlangen, Germany

**Keywords:** Axial length, Choroidal thickness, Contrast sensitivity, Dopamine, Electroretinogram, Ganglion cells, Nearsightedness, Optic disc, Pupil response, Retina

## Abstract

The protective effects of time spent outdoors emphasize the major role of daylight in myopia. Based on the pathophysiology of myopia, the impact of blue light stimulation on the signaling cascade, from melanopsin at the blind spot to clinically relevant biomarkers for myopia, was investigated.

Parameters and site of light stimulation are mainly defined by the photopigment melanopsin, that is sensitive to blue light with a peak wavelength of 480 nm and localized on the intrinsically photosensitive retinal ganglion cells (ipRGC) whose axons converge to the optic disc, corresponding to the physiological blind spot. Blue light at the blind spot (BluSpot) stimulation provides the opportunity to activate the vast majority of ipRGC and avoids additional involvement of rods and cones which may exert incalculable effects on the signaling cascade.

Experimental studies have applied anatomical, histochemical, electrophysiological, imaging, and psychophysical methods to unravel the mode of action of BluSpot stimulation. Results indicate activation of melanopsin, improvement of contrast sensitivity, gain in electrical retinal activity, and increase of choroidal thickness following BluSpot stimulation. Short-term changes of clinically relevant biomarkers lead to the hypothesis that BluSpot stimulation may exert antimyopic effects with long-term application.

## Background

### Myopia

Myopia is the most common eye disorder in the world affecting children and adolescents (Morgan et al. 2021). Progressive myopia is characterized by a disproportionate axial elongation of the eyeball, which impairs distant vision. The progression of myopia increases the risk of severe eye diseases later in life, such as cataract, glaucoma, retinal detachment, and macular degeneration among others (Haarman et al. 2020).

Genetic and environmental factors contribute to the multifactorial nature of progressive myopia (Martínez-Albert et al. 2023; Morgan et al. 2021). Outdoor activity of at least 40 min per day has been shown to be a key factor in reducing myopia incidence and prevalence (Martínez-Albert et al. 2023; Rose et al. 2008; Zhang and Deng 2020). In comparison to other modifiable risk factors such as near work and digital screen time that have a moderate or weak relationship to myopia, whereas near work per se has a substantial impact on myopia development (Biswas et al. 2024; Huang et al. 2015; Karthikeyan et al. 2022), only time outdoors is suggested to have a strong influence on myopia development (Martínez-Albert et al. 2023; Rose et al. 2008). It has been proposed that an increase in light exposure, dopamine release, vitamin D, or the increased depth of field mediate the protective effect of outdoor activity (Cohen et al. 2012; French et al. 2013; Muralidharan et al. 2021; Zhang and Deng 2020).

**Fig. 1:**
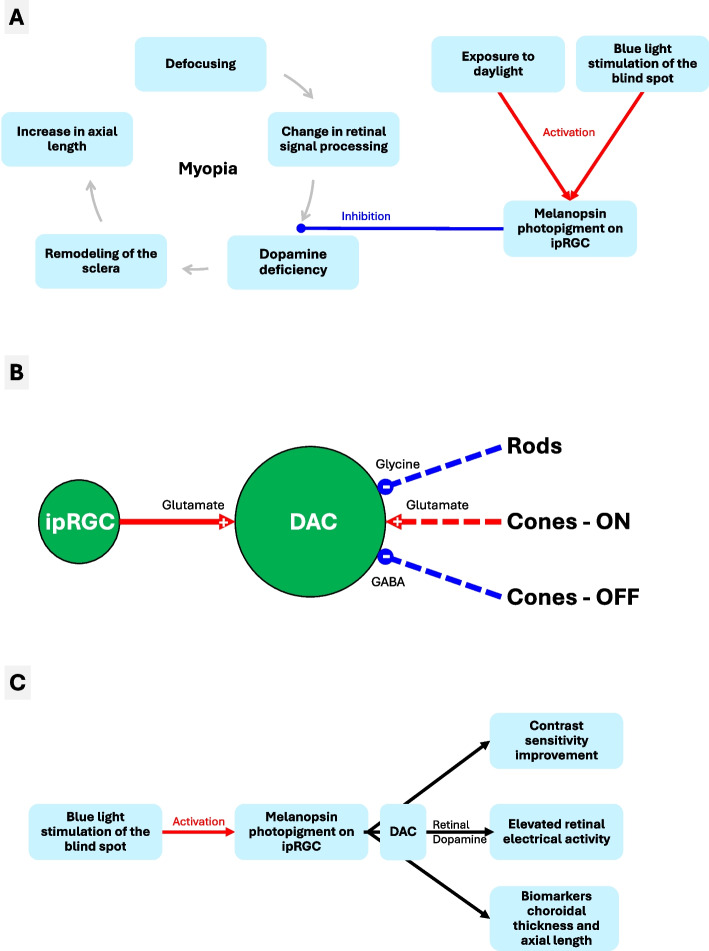
Pathophysiology of myopia and mode of action of blind spot stimulation. **A** Hyperopic defocus significantly alters neuronal signal processing in the retina and reduces dopamine release from amacrine cells (Troilo et al. 2019), resulting in a dopaminergic deficiency that is associated with choroidal thinning. The effects on the sclera are numerous: degradation of collagen, thinning of the sclera, loss of cross-linking resulting in mechanical destabilization. Because of the difference in pressure between the inside and outside of the eye, these factors cause axial elongation of the eye, leading to myopia (Chakraborty et al. 2020; Németh et al. 2021; Troilo et al. 2019). The effect of daylight on the development of axial length is mediated by the neurotransmitter dopamine (Cohen et al. 2012; Muralidharan et al. 2021; Zhang and Deng 2020). Daylight activates the photopigment melanopsin, which is localized in the axons’ membrane of ipRGC. This activation increases the release of dopamine in the retina via an excitatory synaptic contact to dopaminergic amacrine cells. Blue light stimulation of the blind spot is suggested to stimulate melanopsin in the same way as daylight since this site of stimulation offers the possibility of exciting as many melanopsin-containing axons of ipRGC as possible through a relatively small light stimulus. The activation of ipRGC may result in increased dopamine release and inhibition of the pathophysiological signaling cascade of myopia. **B** The dopamine release from DAC in the retina is influenced by excitatory ( +) and inhibitory (-) signaling pathways from different photoreceptors (Newkirk et al. 2013). The activation of the photopigment melanopsin, located on ipRGC, has a direct monosynaptic (solid line) excitatory effect on DAC. Rods and cones indirectly affect the DAC via interneurons, as indicated by the dashed lines. It is assumed that rods inhibit DAC through two synapses by releasing the inhibitory neurotransmitter glycine. Cones can excite DAC through the ON signaling pathway via glutamate and inhibit DAC through the OFF signaling pathway via gamma-aminobutyric acid (GABA). **C** Blue light stimulation of the blind spot activates the melanopsin photopigments on the ipRGC. Various studies are described that have investigated the effect on contrast sensitivity (Schilling et al. 2023), retinal activity (Amorim-de-Sousa et al. 2021; Schilling et al. 2022) and the biomarkers choroidal thickness and axial length (Chakraborty et al. 2024; Ellrich et al. 2023; Hoseini-Yazdi et al. 2024). The possible mechanism of action of blind spot stimulation with blue light via melanopsin-containing ipRGC via retinal dopamine release by DAC on the surrogate markers is shown

Several transmitters and modulators have been suggested to play a role in the regulation of ocular growth, however, it is unclear if their effects originate from the retina (Troilo et al. 2019). The potential roles of monoamines (such as melatonin, serotonin, and epinephrine), the vasoactive intestinal peptide, and glucagon in the retina are still unclear. In contrast to glucagon, intravitreal administration of insulin stimulates eye growth, inducing a myopic shift in otherwise untreated eyes. However, it is unknown and questionable whether insulin can be produced and released in the retina. The so-called light-dopamine theory suggests that sunlight triggers the release of retinal dopamine, which is involved in axial growth regulation (Feldkaemper and Schaeffel 2013; Muralidharan et al. 2021; Zhang and Deng 2020). Intrinsically photosensitive retinal ganglion cells (ipRGC) have been shown to synapse with dopaminergic amacrine cells (DAC) (Newkirk et al. 2013; Sakamoto et al. 2005; Zhang et al. 2008). Considering the potential role of dopamine in refractive development, the involvement of ipRGC and their photopigment melanopsin in myopia’s pathophysiology seems likely (Fig. 1A). Indeed, the pathway connecting ipRGC, dopamine, and refractive development has gained attention recently (Chakraborty et al. 2022; Schaeffel and Swiatczak 2024).

Untreated early childhood eye conditions, for example ptosis and corneal opacity, that significantly impair sharp vision (focusing), trigger myopization (Gee and Tabbara 1988; Huo et al. 2012; Németh et al. 2021; Troilo et al. 2019). These clinical observations have driven the development of experimental models to study the pathophysiology of myopia (Brown et al. 2022; Chakraborty et al. 2020; Németh et al. 2021; Troilo et al. 2019). It is considered that defocus, a disturbance of sharp vision, alters neuronal signaling in the retina and reduces the release of the neurotransmitter dopamine from amacrine cells and promotes the release of all-*trans* retinoic acid (Fig. 1A) (Troilo et al. 2019). This dopaminergic deficiency is associated with a reduction in choroidal thickness, which is acknowledged as a biomarker for myopia (Liu et al. 2021). However, myopia development relies on changes in the sclera, the elastic and mechanically resistant outer layer of the eye globe. The effects on the sclera are manifold and include collagen degradation, thinning of the sclera, loss of cross-linking, and mechanical destabilization. Under these conditions and additionally under the influence of the existing higher intraocular pressure – the difference in pressure between the inside and outside of the eye, the eye undergoes axial elongation, leading to myopia (Fig. 1A) (Chakraborty et al. 2020; Németh et al. 2021; Troilo et al. 2019). Troilo et al. (2019) discussed the potential role of IOP in the development of myopia (Fig. 1A). The scleral changes in experimental myopia development in primates, tree shrews, guinea pigs, and mice are similar to those associated with high myopia in humans (Troilo et al. 2019). There is a restructuring of the scleral extracellular matrix (ECM), a loss of ECM and scleral thinning (Troilo et al. 2019). These alterations are associated with several changes in the mechanical properties of the sclera. Specifically, there are increases in the viscoelasticity and creep rate of the sclera, which make the tissue more extensible so that normal IOP may produce an enlargement of the vitreous chamber (Troilo et al. 2019).

The effect of daylight on the development of axial length is mediated by the neurotransmitter dopamine (Cohen et al. 2012; Muralidharan et al. 2021; Zhang and Deng 2020). Daylight activates the photopigment melanopsin, which is localized in the axons’ membrane of ipRGC. This activation increases the release of dopamine in the retina via an excitatory synaptic contact to DAC. Blue light stimulation at the blind spot (BluSpot) is suggested to stimulate melanopsin in the same way as daylight. This site of stimulation offers the possibility of exciting as many melanopsin-containing axons of ipRGC as possible through a relatively small light stimulus. The activation of ipRGC may result in increased dopamine release and inhibition of the pathophysiological signaling cascade of myopia.

### Dopamine

DAC constitute less than 1% of all amacrine cells in the retina (Troilo et al. 2019; Witkovsky 2004). Depending on light conditions and the circadian rhythm, DAC release dopamine, which interacts pharmacologically with dopaminergic receptors on neighboring cells. Dopamine plays a crucial role in regulating contrast sensitivity, light adaptation, and eye growth (Jackson et al. 2012; Németh et al. 2021).

Animal models have indicated a link between dysregulation of retinal dopamine system and the excessive ocular growth associated with myopia development (Guo et al. 1995; Muralidharan et al. 2021; Thomson et al. 2019). A preclinical study in chicks investigated the relationship between retinal dopamine and lens induced refractive errors (Guo et al. 1995). After two weeks of lens wear, the chicks’ eyes treated with positive lenses were hyperopic, while the eyes treated with negative lenses were myopic. At the same time, in myopic eyes the levels of retinal dopamine and its metabolite 3,4-dihydroxyphenylacetic acid (DOPAC) were reduced compared to control eyes, while in hyperopic eyes the levels of retinal dopamine and DOPAC increased. Intravitreal or topical application of levodopa, the precursor molecule of dopamine, inhibits the development of experimental myopia (Thomson et al. 2019). Topical levodopa remains effective over long-term treatment periods, with its effectiveness enhanced by coadministration with carbidopa to prevent its premature metabolism. Accordingly, dopamine has been suggested to play a key role in experimentally induced myopia in animal models (Troilo et al. 2019), implying the same for the pathophysiology of progressive myopia (Fig. 1A).

The drug methylphenidate inhibits the reuptake of dopamine from the synaptic cleft by the secreting neuron. As a result, methylphenidate increases the concentration of dopamine in the synaptic cleft and is therefore used to treat attention deficit hyperactivity disorder (ADHD) in children. In an experimental study, daily administration of methylphenidate into the vitreous body over a period of one week reduced the development of myopia in laboratory animals by 50% (Karouta et al. 2023). A clinical pilot study investigated the hypothesized effect of methylphenidate on the development of myopia in children over a period of one year (Gurlevik et al. 2021). While ADHD patients treated with methylphenidate showed no changes in the refraction and axial eye length, significant changes in myopia development were observed in the untreated control group. The results from preclinical and clinical studies suggest the hypothesis that methylphenidate may increase the ocular dopamine level and may prevent myopia.

In neurodegenerative Parkinson's disease, the loss of dopaminergic neurons in the basal ganglia of the brain can cause the typical motor syndrome as well as visual symptoms such as reduced contrast sensitivity, indicating a loss of DAC in the retina (Alves et al. 2023). Pharmacological therapy with levodopa, the precursor molecule of dopamine, is particularly effective in the early stages of the disease and also has a positive effect on visual symptoms, confirming that dopamine deficiency is a common cause (Bulens et al. 1987; Hutton et al. 1993).

The release of dopamine from DAC in the retina is influenced by various photoreceptor signaling pathways, both excitatory and inhibitory (Newkirk et al. 2013; Pérez-Fernández et al. 2019). While rods and cones can exert both inhibitory and excitatory effects on DAC, the activation of the photopigment melanopsin, located on ipRGC, has been suggested to excite DAC (Fig. 1B). Therefore, the activation of melanopsin is expected to trigger dopamine release from DAC, although some studies in mice suggest that light induced release of retinal dopamine may be independent of melanopsin phototransduction (Cameron et al. 2009; Munteanu et al. 2018).

### Melanopsin

**Fig. 2:**
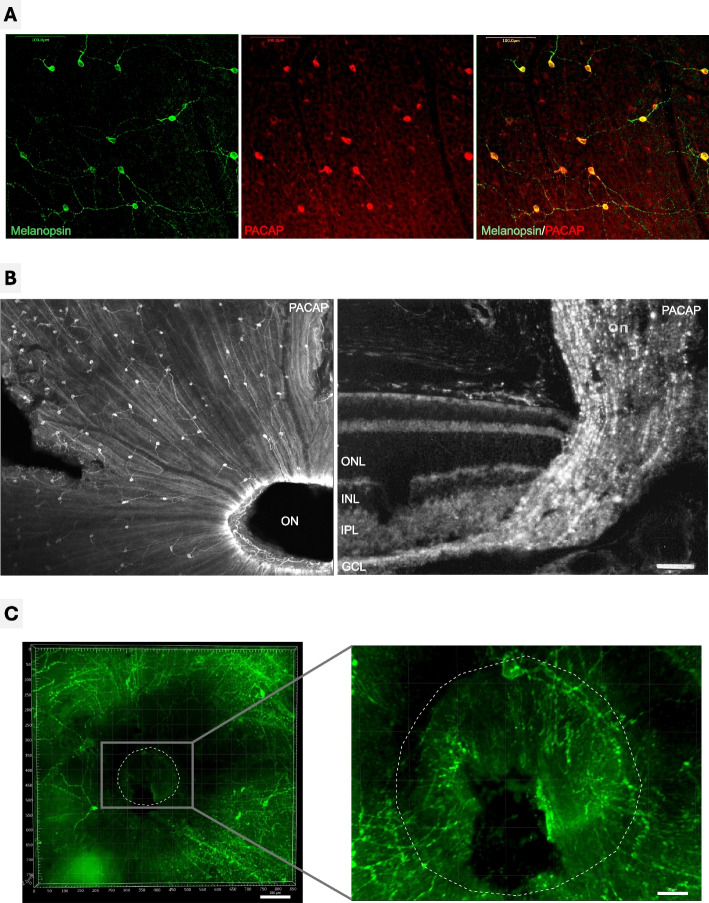
Melanopsin at the optic disc in ipRGC. **A** Melanopsin immunoreactivity is found in ipRGC (left panel) located in the membrane of soma and processes, and in all ipRGC PACAP immunoreactivity can be found primary in the cell cytoplasm and in axons (middle and left panel). Melanopsin is labelled in green, PACAP in red. **B** PACAP, a neurotransmitter and marker for ipRGC is widely distributed in the retina sending axons towards the optic disc (left panel). Since the optic nerve (ON) including the optic disc was cut off, there is no staining. The PACAP containing axons found in the nerve fiber layer reach the optic nerve (right panel) projecting to the brain, when optic disc was preserved. ON = optic nerve, ONL = outer nuclear layer, INL = inner nuclear layer, IPL = inner plexiform layer, GCL = ganglion cell layer. **C** Melanopsin immunoreactive RGC in the mouse retina (left panel) with preserved optic disc. Detection of melanopsin in a 3D reconstruction of the mouse papilla showing melanopsin immunoreactivity in green. The overview shows with indication of the papilla (dashed line) surrounded by melanopsin nerve fibers and cell bodies. In the right panel: Melanopsin immunoreactive RGC in the optic disc of the mouse retina. At higher magnification (than the left panel) the papilla is shown in which melanopsin nerve fibers are observed to be leaving the eye. The dashed line indicates the optic disc margin. Melanopsin immunoreactivity in green shows abundant axons within the optic disc as well as some cell bodies with melanopsin at the edge of the optic disc

Melanopsin is expressed in membranes of dendrites, axons and cell bodies of human ipRGC (Fig. 2A), which account for approximately 0.7% of all retinal ganglion cells (Hannibal et al. 2017; Nasir-Ahmad et al. 2019). Blue light with a peak wavelength of approximately 480 nm activates melanopsin and subsequently excites ipRGC (Bailes and Lucas 2013). Preclinical studies have shown that action potentials propagate retrogradely towards the ipRGC cell body (Brill-Weil et al. 2024; Nath et al. 2024; Prigge et al. 2016). ipRGC have synaptic access to DAC via branches of their nerve fibers (Nath et al. 2024; Prigge et al. 2016). In an in vitro experiment in mice, it was shown that blue light stimulation of the optic disc evokes phasic spiking activity of ipRGC and DAC (Brill-Weil et al. 2024; Nath et al. 2024). Accordingly, in chicks exposed to blue light for 30 min in only one eye while the contralateral eye was covered, the vitreous body of the stimulated eye showed an increase in DOPAC (Wang et al. 2018).

### Blind spot

The axons of the retinal ganglion cells exit the eye at the optic disc and form the optic nerve. In this anatomical region, there are no rods or cones, resulting in a physiological absolute visual field loss known as the blind spot. Anatomically, the blind spot is located approximately 15° nasal to the fovea centralis, the center of the eye's sharpest vision. Retinal ganglion cells’ axons, including ipRGC, converge at the optic disc whereas melanopsin expressing cells exclusively also express pituitary adenylate cyclase-activating polypeptide (PACAP) (Fig. 2A, B) (Esquiva et al. 2016; Fahrenkrug et al. 2004). Melanopsin can be detected in the axon membrane in the initial sections of the optic nerve (Fig. 2C) but not beyond the lamina cribrosa (Brill-Weil et al. 2024; Esquiva et al. 2016; Fahrenkrug et al. 2004), qualifying this location for blue light stimulation. These axons of ipRGC converge in the optic disc, also called the physiological blind spot. The notable density of melanopsin-carrying ipRGC axons at the optic disc (Fig. 2) allows for the excitation of the vast majority of ipRGC with a relatively small area of blue light stimulus (Fig. 1A). There are no rods and cones in the optic disc. Simultaneously, light stimulation at the blind spot without additional activation of rods and cones prevents confounding effects on DAC through other photoreceptive pathways and coincides with the physiological property of the blind spot, allowing for blue light stimulation that is barely perceptible to the patient.

Addressing the question of how blind spot stimulation with blue light can alter clinically relevant biomarkers such as choroidal thickness and axial length, several surrogate markers were investigated in humans. To elucidate the mode of action of BluSpot stimulation, experimental studies applied a range of methodologies, including pupillography, psychophysical methods to measure contrast sensitivity, electroretinography (ERG) to examine retinal activity, optical coherence tomography (OCT) for assessment of choroidal thickness and optical biometry for axial length measurement (Fig. 1C). Specifically, the following questions were investigated in humans:

- Does BluSpot stimulation activate melanopsin?
- Is contrast sensitivity improved after BluSpot stimulation as with application of dopamine agonists?
- Are retinal processes related to dopamine release upregulated after BluSpot stimulation?
- How does BluSpot stimulation modulate choroidal thickness and axial length?

The outcome of the experimental studies and the effect of BluSpot stimulation under specific light parameters are depicted in Table 1.

**Table 1:**
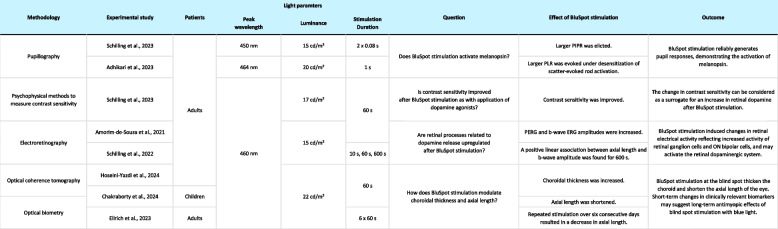
Summary of the results of the experimental studies in humans using BluSpot stimulation

## Mode of action

### Pupil response

The first question about melanopsin activation was assessed with pupil responses. One way to quantify melanopsin activation of ipRGC in humans is to measure the pupil light response (PLR) and the post-illumination pupil response (PIPR) which has been shown to be specifically reflective for melanopsin (Adhikari et al. 2015). Since the peak sensitivity of melanopsin is approximately 480 nm (Bailes and Lucas 2013), a suitable active control stimulation is red light with a peak wavelength over 600 nm.

BluSpot stimulation elicited a larger PIPR than red light control stimulation in 15 healthy volunteers (Schilling et al. 2023). In a subsequent study with six healthy volunteers, the PLR evoked by BluSpot stimulation was significantly larger than with red light control stimulation even under desensitization of scatter-evoked rod activation by a cyan background (Adhikari et al. 2023). The experimental data suggest that BluSpot stimulation reliably generates pupil responses, demonstrating the ability of this stimulation to activate melanopsin.

### Contrast sensitivity

Genetically modified mice, deficient in melanopsin, have reduced contrast sensitivity (Schmidt et al. 2014), suggesting that ipRGC are involved in its modulation. Furthermore, there is evidence that in addition to transmitters such as serotonin (Sato et al. 2020), acetylcholine (Boucart et al. 2015) and GABA (Harris and Phillipson 1995), dopamine contributes to contrast sensitivity. In healthy adults, administration of levodopa and nomifensine, both dopamine agonists, improve contrast sensitivity. Correspondingly, in patients with Parkinson's disease, where the dopaminergic neurons are degenerated, contrast sensitivity is reduced (Alves et al. 2023).

In a study with 32 volunteers, two psychophysical experiments showed that contrast sensitivity was improved 20 min after BluSpot stimulation. This change in contrast sensitivity can be considered as a surrogate for an increase in retinal dopamine after BluSpot stimulation (Schilling et al. 2023).

### Electroretinography

BluSpot stimulation induced changes in retinal electrical activity (Amorim-de-Sousa et al. 2021; Schilling et al. 2022). Specifically, the changes appeared in the light-adapted b-wave of the ERG and P50-N95 of Pattern ERG (PERG) amplitudes. The PERG measures the retinal response to chessboard pattern visual stimuli to assess the retinal ganglion cell activity. The b-wave of the light-adapted ERG is a positive deflection that reflects the activity of the retina to a light stimulus, with input from ON bipolar cells associated with the BluSpot stimulus. Changes in PERG and photopic b-wave ERG amplitudes were increased 20 min after BluSpot stimulation in myopic eyes, reflecting increased activity of retinal ganglion cells and ON bipolar cells (Amorim-de-Sousa et al. 2021).

In another study of the b-wave ERG, the effect of different stimulus durations was investigated, and it was concluded that even 1 min of BluSpot stimulation may activate the retinal dopaminergic system in myopes, with longer stimulation inducing increased b-wave response (Schilling et al. 2022). With a stimulation duration of 10 min on the blind spot, a positive linear association between axial length of the eye and change in b-wave amplitude was found. This means that longer myopic eyes exhibited a trend to be more sensitive of increased retinal electrical activity after BluSpot stimulation.

### Choroidal thickness and axial length

The current understanding of myopia's pathophysiology suggests that a decrease in dopamine release in the retina is associated with a reduction in choroidal thickness (Fig. 1A) (Chakraborty et al. 2020; Németh et al. 2021; Troilo et al. 2019). Several clinical studies have investigated the treatment of progressive myopia with special spectacles, contact lenses, or atropine eyedrops concluding that a short-term increase in choroidal thickness after one to four weeks of therapy can predict long-term reduction of eye elongation in myopic children after 6 to 24 months (Chun et al. 2021; Li et al. 2019; Ye et al. 2020). Consequently, an increase in choroidal thickness has been proposed as a clinical biomarker for potential therapies for myopia (Ostrin et al. 2023; Read et al. 2019). The potential relationship between an increase in dopamine and an increase in choroidal thickness was investigated in animal experiments (Mathis et al. 2023). Stimulation with flickering light over a period of two hours resulted in an increase in both retinal dopamine concentration and choroidal thickness.

Young adults were illuminated by light of different wavelengths for one hour while only the right eye was exposed to a hyperopic defocus, an optical stimulus known to result in choroidal thinning and axial elongation. Full-field blue light exposure was found to inhibit the effects of hyperopic defocus and led to a reduction in the axial length of both eyes, whereas an increase in axial length was found after red and green light accompanied by significant decreases in choroidal thickness (Thakur et al. 2021).

In contrast to full-field stimulation, local BluSpot stimulation avoids possible negative influences from rods and cones. Such selective local stimulation is suggested to produce similar or even larger effects than full-field stimulation. Accordingly, twenty adult volunteers showed an increase in choroidal thickness within one hour after a single one-minute BluSpot stimulation compared to control conditions without stimulation or with red light stimulation at the blind spot (Hoseini-Yazdi et al. 2024). Repeated BluSpot stimulation over six consecutive days resulted in a decrease in axial length of 16.6 ± 7.9 µm in myopic adults (*n* = 5), while axial length remained unchanged in emmetropes (*n* = 5) (Ellrich et al. 2023). In a study involving 10 emmetropic and 10 myopic children, the subfoveal choroidal thickness increased after 1-min BluSpot stimulation compared to red light stimulation at the blind spot (Chakraborty et al. 2024). The choroidal thickness continued to increase over time, reaching an increase of 6.2 µm after 60 min. Consistently, the axial length was shortened after BluSpot stimulation compared to active red light control.

### Blue light stimulation

Based on results from animal studies and the relationship between melanopsin activation, dopamine release, choroidal thickness increase and axial length reduction, a digital application (app) is developed for use in humans that allows reliable BluSpot stimulation (MyopiaX®, Dopavision GmbH). The app runs on a standard cell phone, which is inserted into a virtual reality headset to stimulate the blind spot in both eyes. The BluSpot stimulation is applied at intensities and view times that exclude potential damage to the photoreceptors (O’Hagan et al. 2016). The circular area of the blue light stimulus is slightly smaller than the blind spot and is adapted in location to the individual anatomy of the retina, resulting in a quasi-invisible stimulation for each patient. To ensure successful treatment, children are instructed to look at a specific point on the mobile phone screen with their head held straight. A Bluetooth game controller is used to control a virtual reality game during the intervention to stabilize gaze and reliably stimulate the BluSpot. The app was developed specifically for children and consists of several age-appropriate, interactive tasks to maintain engagement.

Randomized clinical trials are currently underway to investigate the effects of daily BluSpot stimulation lasting a few minutes on the progression of myopia and the associated choroidal thickness in myopic children (NCT04967287 at ClinicalTrials.gov; ACTRN12623000928617 at ANZCTR.org.au).

## Conclusions

Stimulating the blind spot with blue light is based on the light-dopamine theory and the clinical finding that daylight can protect against the development of progressive myopia. The type and location of light stimulation are defined by the photopigment melanopsin, which is highly sensitive to blue light and is localized on ipRGC. The axons of ipRGC converge towards the blind spot. The stimulation site at the blind spot offers the possibility of activating a large number of ipRGC and DAC while avoiding additional excitation of rods and cones, which can inhibit dopamine release. This proposed dopaminergic effect via BluSpot stimulation may also be triggered invisibly for the patient since no rods and cones are located in the blind spot.

Exploring the mechanism of action, BluSpot stimulation at the blind spot has been shown to evoke pupil response, improve contrast sensitivity, increase electrical retinal activity, thicken the choroid and shorten the axial length of the eye. Short-term changes in clinically relevant biomarkers may suggest long-term antimyopic effects of blind spot stimulation with blue light.

## Data Availability

Not applicable. No datasets were generated or analyzed.

## Abbreviations

App: Application
BluSpot: Blue light at the blind spot
DAC: Dopaminergic amacrine cells
DOPAC: 3,4-Dihydroxyphenylacetic acid
ECM: Extracellular matrix
ipRGC: Intrinsically photosensitive retinal ganglion cells
OCT: Optical coherence tomography
PACAP: Pituitary adenylate cyclase-activating polypeptide
PIPR: Post-illumination pupil response
PLR: Pupil light response
